# Expression and Immunogenicity of Recombinant African Swine Fever Virus Proteins Using the Semliki Forest Virus

**DOI:** 10.3389/fvets.2022.870009

**Published:** 2022-05-09

**Authors:** Niran Fang, Bin Yang, Ting Xu, Yanpeng Li, Huimin Li, Hanghui Zheng, Aiguo Zhang, Ruiai Chen

**Affiliations:** ^1^Zhaoqing Institute of Biotechnology Co. Ltd., Zhaoqing, China; ^2^College of Veterinary Medicine, South China Agricultural University, Guangzhou, China; ^3^Zhaoqing DaHuaNong Biology Medicine Co. Ltd., Zhaoqing, China; ^4^Jinggangshan University, Jinggangshan, China; ^5^Henan University of Animal Husbandry and Economy, Zhengzhou, China

**Keywords:** African swine fever virus, Semliki Forest virus, replication-defective viral particles, immunogenicity, cellular immune response

## Abstract

African swine fever virus (ASFV) is a large DNA virus belonging to the Asfarviridae family that damages the immune system of pigs, resulting in the death or slaughter of millions of animals worldwide. Recent modern techniques in ASFV vaccination have highlighted the potential of viral replicon particles (RPs), which can efficiently express foreign proteins and induce robust cellular and humoral immune responses compared with the existing vaccines. In this study, we established a Semliki Forest virus (SFV) vector by producing replication-defective viral particles. This vector was used to deliver RPs expressing ASFV antigens. SFV-RPs expressing ASFV p32 (SFV-p32) and p54 (SFV-p54) were tested in baby hamster kidney (BHK-21) cells. Proteins expression was evaluated via western blotting and indirect immunofluorescence, while immunogenicity was evaluated in BALB/c mice. The resulting RPs exhibited high levels of protein expression and elicited robust humoral and cellular immune responses.

## Introduction

African swine fever virus (ASFV) infection affects domestic pigs and causes an acute, febrile, and highly infectious disease. No effective vaccine against ASFV exists to date, and once the infection is detected, the animals must be slaughtered, imposing a large economic burden on the swine industry. ASFV was first observed in Europe in 1957 and spread in Portugal in 1960 wherefrom the virus became established in the Iberian Peninsula and caused outbreaks across Western Europe, the Caribbean, and Brazil. Although ASFV was almost eradicated outside of Africa by the mid-1990s, in 2007 it was detected in Georgia wherefrom it spread throughout Eastern Asia and Eastern Europe, causing large-scale global economic losses. Before 2018, there were no reported cases of ASFV in China, but the government continued to monitor for the disease. In 2013, China joined the ASFV global alliance to strengthen the prevention and control of the disease. This initiated fundamental research efforts with the ultimate goal of developing effective vaccines against ASFV.

In the earliest ASFV studies, attenuated viruses were found to protect from the disease, but their efficacy in the field was not adequate. In recent years, there has been a rapid drive to develop platforms for the expression of a variety of heterologous proteins in animal cells, and there are now many methods and vector expression platforms available for the expression of different antigens. Vector expression platforms based on nucleic acids represent important new approaches for vaccine development, and DNA vaccines have the added advantage of expressing heterologous DNA sequences (1, 2). Recent improvements in vector systems have facilitated the development of virus-based mRNA vaccines (2, 3). RNA replicon vaccines are based on a vector platform derived from the Semliki Forest virus (SFV), which can replicate autonomously and induce systemic, cellular, and mucosal immunity (4). Murgia et al. (5) has described an alphavirus vector carrying replicon particle (RP)-30 and RP-54, using Venezuelan equine encephalitis virus (VEEV) rather than SFV, and with Vero cells rather than BHK-21 cells, which helped establish humoral immunity in swine.

cDNA coding for a variety of viral proteins involved in ASFV infection, such as p32 and p54, can be cloned into the SFV vector. The p32 protein, which has a molecular weight of ~30 kDa and is expressed in the early stages of viral infection, has a good level of antigenicity and can induce a robust immune response. A p32-specific blocking antibody inhibits the internalization of the virus, indicating that this viral component is also involved in the viral entry into cells (6). p54 is a type I transmembrane protein that crosses the inner envelope of the virus particle and forms a homodimer via a cysteine bridge (7). p54 helps the virus to bind to target cells (8) and stimulates the host to produce p54-specific antibodies. In ASFV-infected cells, p54 binds to the endoplasmic reticulum and uses its membrane to form the viral particle envelope. A special cross-reaction between p54 and the dynein light chain (DLC8) mediates intracellular transport of the virus, suggesting that p54 plays an important role in viral uptake and processing (9).

In a previous study, an SFV vector coding for foreign proteins, as well as a pSFV-helper1, was used to encode the SFV structural proteins and transcribe them into mRNA *in vitro*, these recombinant RNAs were electroporated into baby hamster kidney (BHK)-21 cells to express high levels of heterologous proteins (10). In the SFV replicon vector, SFV structural genes can be replaced with heterologous genes of interest, generating a self replicating RNA that can efficiently express the heterologous genes. Replicon RNA supplies the non-structural genes, which contain the packaging signal. The viral structural genes are provided by the helper RNA, which lacks part of the non-structural genes, including the packaging signal. Therefore, only the replicon RNA retains the packaging signal, and it is packaged into RPs through the expression of the structural genes supplied by the helper RNA. The resulting RPs can infect mammalian cells but only undergo one round of replication (10, 11). The alphavirus replicon system has been used to develop effective influenza RP vaccines, which have been evaluated in chickens, pigs, and humans and elicited complete protection against lethal avian influenza (4, 12). Compared to conventional DNA vaccines, the replicon RNA system is self-amplifying, boosting cellular and humoral responses (4). The co-expression of genes encoding Ebola and Lassa virus-specific glycoproteins was used to produce an RP vaccine that protects guinea pigs against Ebola and Lassa viral challenges, indicating that RP vaccines may protect against multiple diseases (13). Another advantage of RP vaccines is that alphaviruses may infect many different cell lines, indicating that cells, such as Chinese hamster ovary, primary chicken embryo fibroblast, duck embryo fibroblast, or human embryonic kidney (HEK) 293 (or 293T) cells, which can yield different amounts of RPs, may be used in commercial vaccine production (4).

Here we aimed to establish an SFV vector by producing replication-defective viral particles and utilized it to deliver RPs expressing ASFV antigens. SFV-RPs expressing ASFV p32 (SFV-p32) and p54 (SFV-p54) were tested in baby hamster kidney (BHK-21) cells. Proteins expression was evaluated via western blotting and indirect immunofluorescence, while immunogenicity was evaluated in BALB/c mice.

## Methods and Materials

### Plasmids, Cells, and Animals

pSFVCs-LacZ and pSFV-helper1 (plasmid numbers #92076 and #92073, respectively) were purchased from Addgene (Watertown, Massachusetts, USA). Fifteen 5-week-old SPF BALB/c mice were purchased from the Animal Experiment Center of Southern Medical University (Guangzhou, People's Republic of China), and raised in individually ventilated cages within the SPF animal house of Zhaoqing DaHuaNong Biology Medicine Co. Ltd. (Zhaoqing, People's Republic of China). The animal study was reviewed and approved by Animal Welfare and Ethical Censor Committee.

### Construction of SFV Vector Plasmids

**Figure 1:**
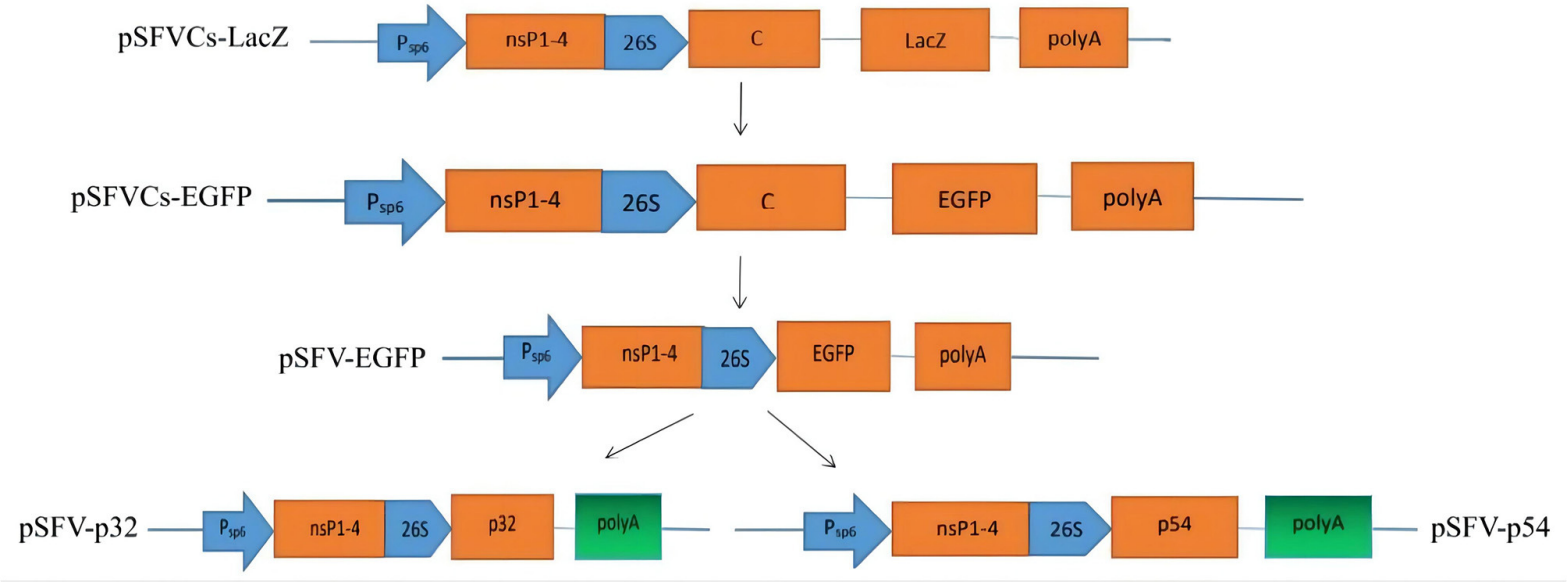
SFV expression vector system. We used the plasmid pSFVCs-LacZ, which contains the non-structural genome of SFV. To insert heterologous genes into the pSFVCs-LacZ plasmid, LacZ was removed via BamHI restriction endonuclease, EGFP- and p32-, p54-coding ASFV genes were inserted into the vector plasmid. We obtained pSFVCs-EGFP, pSFV-EGFP, pSFV-p32, and pSFV-p54.

On an SFV vector platform, using the pSFVCs-LacZ containing the non-structural genome of SFV (Figure 1). The LacZ-encoding gene was removed using a BamHI restriction endonuclease to insert a heterologous gene into pSFVCs-LacZ. Enhanced green fluorescent protein (EGFP)- and ASFV p32- or p54-encoding sequences were inserted into the vector plasmid to establish pSFVCs-EGFP, pSFV-EGFP, pSFV-p32, and pSFV-p54 (Figure 1) based on GenBank sequences for p32 and p54 (accession no. NP_042786 and NP_042818). The above heterologous proteins were inserted downstream of the 26S promoter.

### SFVCs-RP Generation

To obtain SFVCs-RP, the SFV expression vector plasmids described above were linearized using SpeI, then transcribed *in vitro* using the mMESSAGE mMACHINE™ SP6 (Thermo Fisher Scientific, Waltham, MA, USA). pSFVCs-LacZ RNA and pSFVCs-EGFP RNA were electroporated into BHK-21 cells with pSFV-helper1 RNA at 100 V for 25 ms, respectively. BHK-21 cells were cultured in 1% fetal bovine serum (FBS)-containing Dulbecco's Modified Eagle Medium (Gibco) at 37°C until the cells adhered, then the medium was changed to serum-free medium. The viral particles were collected from the supernatant by centrifugation 36 h after co-electroporation, then filtered through a 0.22 μm Millex-GP filter to generate SFVCs-LacZ and SFVCs-EGFP RPs. β-galactosidase was detected using *in situ* β-galactosidase Staining Kit (Beyotime, Shanghai, China), and EGFP was detected using a fluorescence microscope.

### SFV-RP Generation

To avoid interference from the fusion capsid protein during the specific expression of the target protein in the follow-up animal experiments, we removed the viral capsid protein and constructed the replicon plasmid pSFV-EGFP via homologous recombination using the pEASY^®^-Basic Seamless Cloning and Assembly Kit (TransGen Biotech, Beijing, China). SFV-EGFP RP was then obtained using the above-described method for SFVCs-RP. Fluorescence signal intensity was compared with SFVCs-RPs 24 h after infection.

### ASFV SFV-RP Generation

To develop the plasmids expressing ASFV p32 and p54, we constructed the replicon plasmids pSFV-p32 and pSFV-p54 via homologous recombination using the pEASY^®^-Basic Seamless Cloning and Assembly Kit and pSFV-EGFP as described above.

pSFV-p32 and pSFV-p54 were linearized using SpeI and SpaI, respectively, and transcribed *in vitro* using the mMESSAGE mMACHINE™ SP6 (Thermo Fisher Scientific). They were then electroporated into BHK-21 cells with the helper RNA at 100 V for 25 ms. SFV-p32 and SFV-p54 RPs were collected using the same method described above.

### ASFV SFV-RP Viral Titers and Detection of Infected BHK-21 Cells

The viral supernatant was collected by centrifugation, every 6 h starting from 24 h after the replicon RNA electroporation into BHK-21 cells with helper RNA. Then, 100 μL dilutions of viral samples were added to 96-well plates containing BHK-21 cell monolayers. Following 60 h incubation at 37°C under 5% CO_2_, viruses were detected based on the cytopathogenic effect (CPE) and immunofluorescence (IFA). ASFV SFV-RP titration was calculated using the Spearman-Karber method.

### IFA

SFV-p32 and SFV-p54 viral particles were collected every 6 h starting from 24 h after co-electroporation. Cells were cultured in plates with particles for 60 h and washed with phosphate-buffered saline containing Tween (PBST) three times, fixed with formaldehyde for 10 min at 4°C, then washed with PBST and permeabilized with 0.1% Triton X-100. They were then incubated for 2 h at 37°C with 5% bovine serum albumin (BSA), followed by washing and 1 h incubation at 37°C with an anti-ASFV serum antibody (Zhaoqing DaHuaNong Biology Medicine Co. Ltd., Zhaoqing, People's Republic of China) diluted at 1:2500. The samples were then washed again and incubated for 1 h at 37°C with IFTC-conjugated AffiniPure Goat Anti-Swine IgG (H + L) (Jackson ImmunoResearch) diluted at 1:600 as the secondary antibody. They were then washed three times and observed under a fluorescence microscope.

### Western Blotting

BHK-21 cells infected with SFV-p32 and SFV-p54 (RPs) for 36 h were collected and lysed on ice for 5 min. The lysate was mixed with loading buffer (5 ×) and boiled for 10 min. The mixture was loaded onto 12% polyacrylamide gels, electrophoresed for 120 min at 80 V, then transferred to a polyvinylidene difluoride membrane via electroblotting for 90 min. The membranes were blocked with 5% BSA for 1 h at room temperature, then washed three times with PBST for 10 min each. They were incubated overnight at 4°C with an anti-ASFV serum antibody diluted at 1:1,000, then washed and incubated for 1 h at 37°C with a secondary goat anti-swine IgG (H+L) conjugated with horseradish peroxidase (ABclonal) diluted at 1:5,000. Finally, the membranes were washed three times and visualized using a FUSION FX EDGE SPECTRA imaging system (VILBER, Collégien, France).

### ASFV SFV-RP Immunization

Five-week-old BALB/c mice were immunized three times via intramuscular injections of SFV-32 RPs, SFV-54 RPs, or PBS in the lateral thigh (*n* = 5/group). Each mouse was immunized at 2-week intervals at the same dose (0.2 mL) [The SFV-p32 RP titer in BHK-21 cells was 10^3.0^ median tissue culture infectious dose (TCID_50_)/0.1 mL, and the SFV-p54 RPs titer was 10^3.5^ TCID_50_/0.1 mL]. Blood samples were collected from the canthus before the first immunization and 1 week after each immunization and stored at −80°C. Mice were monitored and weighed daily.

### Enzyme-Linked Immunosorbent Assay

ELISA for detecting anti-p32 antibody IgG was performed with the serum samples described above using the ASFV p32 Antibodies Test Kit (sandwich ELISA) according to the manufacturer's instructions (Zhaoqing DaHuaNong Biology Medicine Co. Ltd., Zhaoqing, China). Microplates were coated with the ASFV p32 antigen and the serum samples were added, followed by HRP-labeled ASFV antigen. After washing the plate, the substrate was added, and the blue color was observed. The reaction was then terminated, giving a yellow color. The specific absorption peak at 450 nm was observed using a Spectrophotometer Multiskan Skyhigh (Thermo Scientific).

ELISA for detecting anti-p54 antibody IgG was performed using AsurDxTM ASFV p54 Antibody Test Kit (BioStone Animal Health, Dallas, TX, USA). The microplates were coated with the ASFV p54 antigen. The serum samples were added and the microplates were washed five times. An anti-ASF monoclonal antibody (mAb)-biotin corjugate and HRP-tagged streptavidin were added. After incubation for 30 min at 37°C, the plate was washed and the substrate was added. The color change was observed and the reaction was terminated, which caused another color change (blue to yellow). The specific absorption peak at 450 nm was measured using a Spectrophotometer Multiskan Skyhigh (Thermo Scientific). Three parallel experiments were performed for each sample.

### Enzyme-Linked Immune Absorbent Spot Assay

Mice were euthanized 2 weeks after the third immunization. Lymphocytes were aseptically isolated from the spleens for cellular immunity experiments. Interferon (IFN)-γ and interleukin (IL)-4 expressions were measured using anti-mouse IFN-γ- and anti-mouse IL-4-precoated ELISPOT kits (Dakewe Biotech Co., Ltd., Shenzhen, China). The synthesized polypeptides were p32-1, DFNKV IRAHN FIQTI; p32-2, IRAHN FIQTI HGTPL; p54, ENLRQ RNTYT HKDLE (Sangon Bioengineering Co., Ltd., Shanghai, China). Each group was assigned a positive control (10 μL PHA with resuspended cells), a negative control (without stimulant but with resuspended cells), and a background negative control (only the culture medium). Mice immunized with SFV-p32 RP (with stimulant polypeptides p32-1 and p32-2, the final concentration is 60 μg/mL), and SFV-p54 RP (with stimulant polypeptide p54, the final concentration is 60 μg/mL) (five mice each) were used. An immunospot image analyzer from the Dakewe Biotech Co., Ltd., Shenzhen, China was used.

### Statistical Analysis

SPSS software (IBM SPSS Statistics for Windows, Version 20.0, IBM Corporation, Armonk, NY, USA) was used for statistical analysis. GraphPad Prism (version 9.2, GraphPad Software Inc., CA, USA) was used to analyze data. Significance levels were represented by *P*-values (ns, non-significant; *P < 0.05; **P < 0.01; ***P < 0.001).

## Results

### SFVCs-RP Production

**Figure 2:**
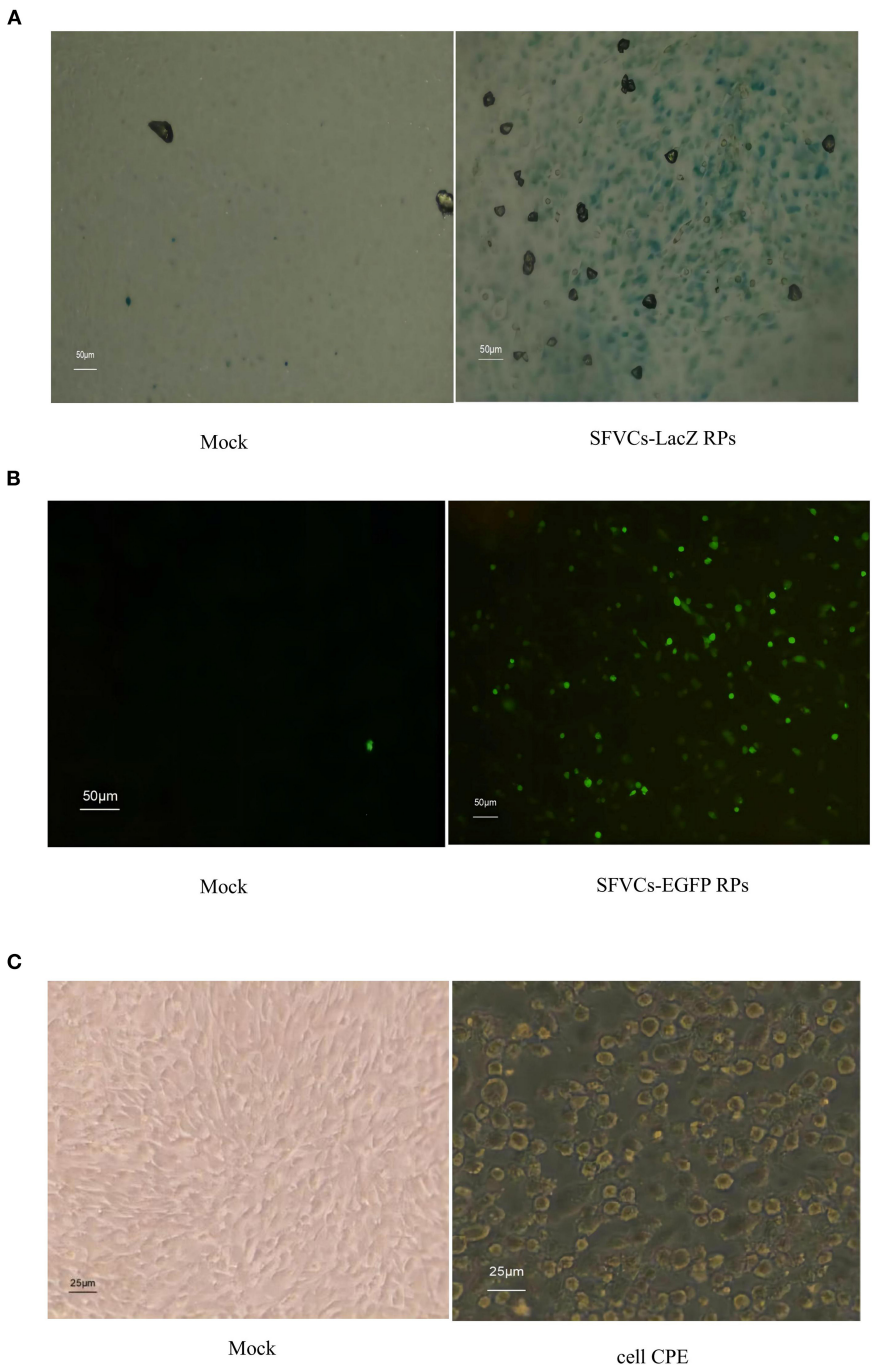
Expression of heterologous proteins in BHK-21 cells with SFVCs-RP infected. **(A)** β-galactosidase expression (×200). **(B)** EGFP expression (×200). **(C)** Cells were infected with SFVCs-EGFP RPs (×400) for 24 h. CPE was evaluated.

The SFVCs-LacZ and SFVCs-EGFP RPs were successfully packaged. The blue color due to β-galactosidase was observed in 80–90% of cells, while few control cells were stained (Figure 2A). EGFP signal was detected using a fluorescence microscope, and more than half of the cells emitted green fluorescence (Figure 2B). Cells infected for 24 h with SFVCs-LacZ and SFVCs-EGFP RPs showed evident CPE (Figure 2C).

### SFV-RP Production

**Figure 3:**
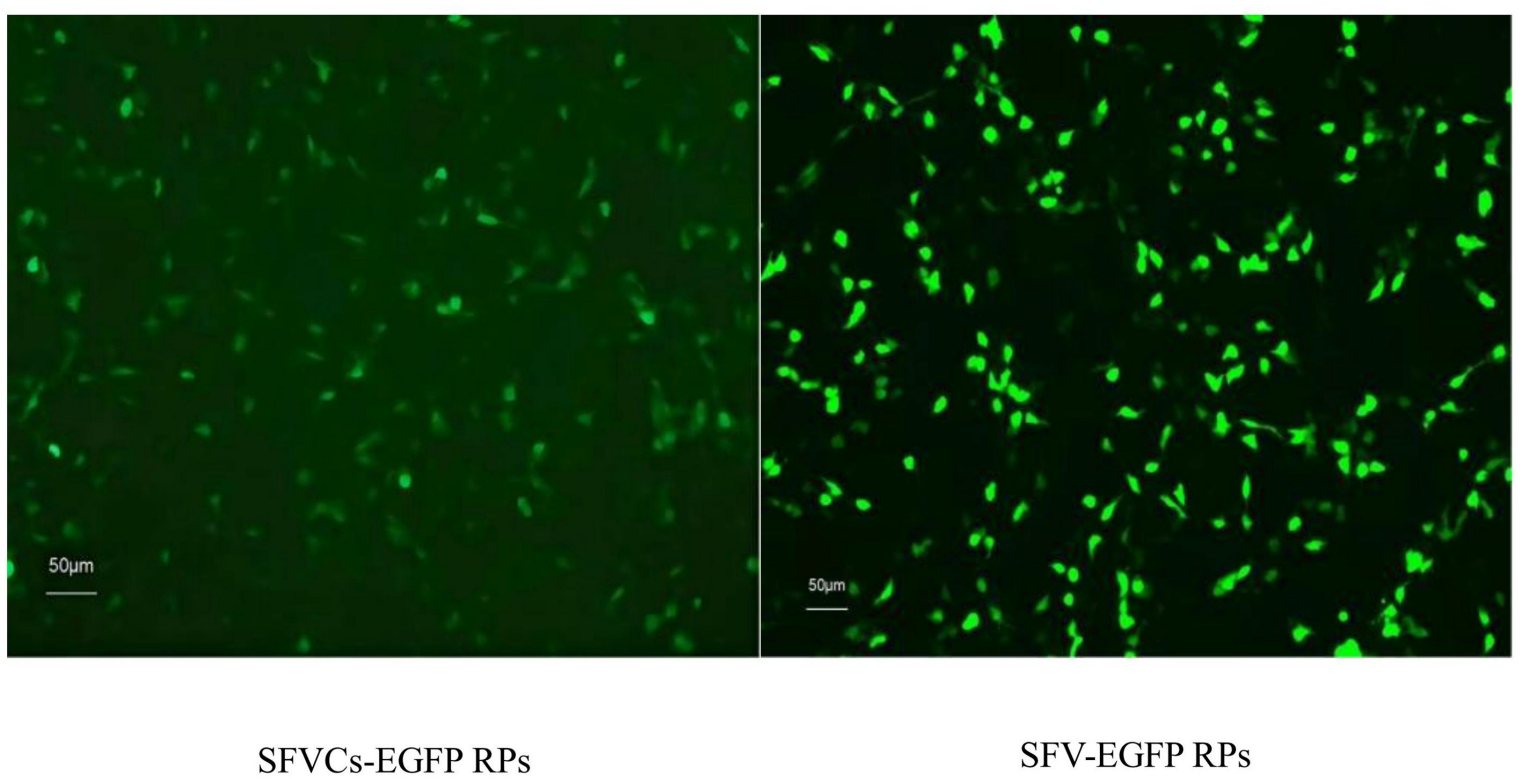
Fluorescence signal intensity was compared after removing the viral capsid protein. BHK-21 cells were infected with SFVCs-EGFP and SFV-EGFP RPs for 24 h, and fluorescence signal intensities were compared (×200).

SFV-EGFP RPs were successfully packaged. BHK-21 cells were infected with SFV-EGFP RPs and SFVCs-EGFP RPs for 24 h, and fluorescence signal intensities showed that the expression of EGFP in the former was not decreased compared with the latter (Figure 3), confirming that the SFV vector could efficiently express foreign genes.

### ASFV SFV-RP Production

EGFP in the pSFV-EGFP was replaced with ASFV antigens p32 and p54 and electroporated into BHK-21 cells. SFV-p32 and SFV-p54 RPs were successfully packaged.

### IFA of Infected BHK-21 Cells and Viral Growth of ASFV SFV-RPs

**Figure 4:**
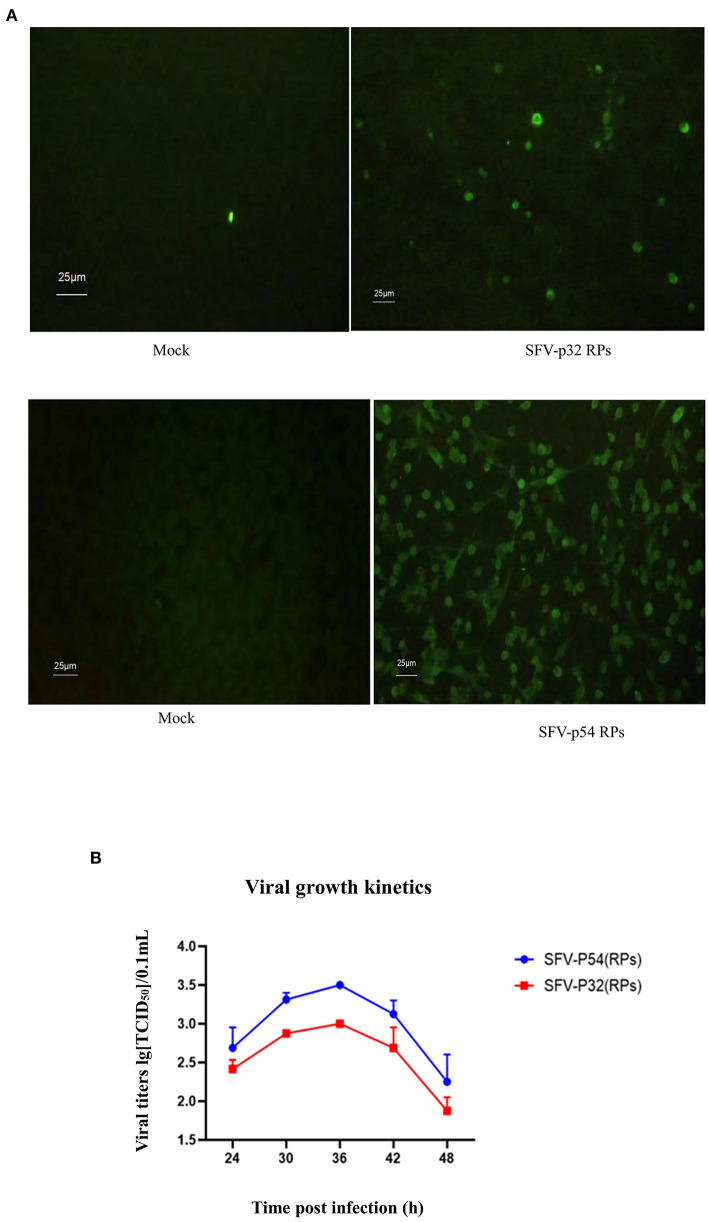
IFA and viral growth of ASFV SFV-RPs. **(A)** IFA of ASFV SFV-RPs infected BHK-21 cells after 60 h (×400). **(B)** Viral growth in ASFV SFV-RPs-infected BHK-21 cells.

BHK-21 cells infected with SFV-p32 and SFV-p54 RPs for 60 h were detected using IFA. The results showed that infected cells exhibited green fluorescence and CPE at a suitable viral titer (Figure 4A).

The titers of SFV-p32 and SFV-p54 RPs in BHK-21 cells continuously increased from 24 to 36 h post-infection and peaked (10^3.0^ TCID_50_/0.1 mL,10^3.5^ TCID_50_/0.1 mL, respectively) 36 h post-infection (Figure 4B). The subsequent decrease was related to cell death.

### ASFV SFV-RP Western Blotting

**Figure 5:**
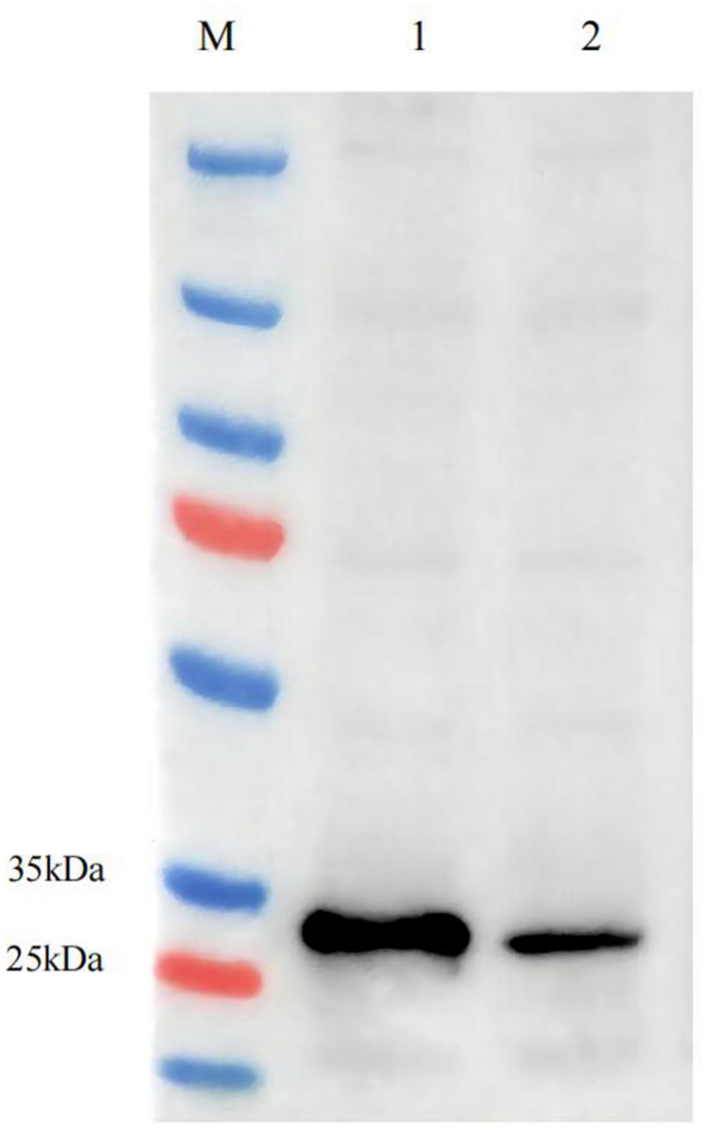
Western blotting of ASFV SFV-RPs. ASFV p32 expression in SFV-p32 RP-infected BHK-21 cells (Lane 1), ASFV p54 expression in SFV-p54 RP-infected BHK-21 cells (Lane 2).

To test heterologous p32 and p54 protein expression, cells were lysed and analyzed via western blotting. The bands at 30 kDa confirmed successful packaging of the SFV-p32 and SFV-p54 RPs and efficient infection of BHK-21 cells (Figure 5).

### Mouse Weight Changes After Immunization

**Figure 6:**
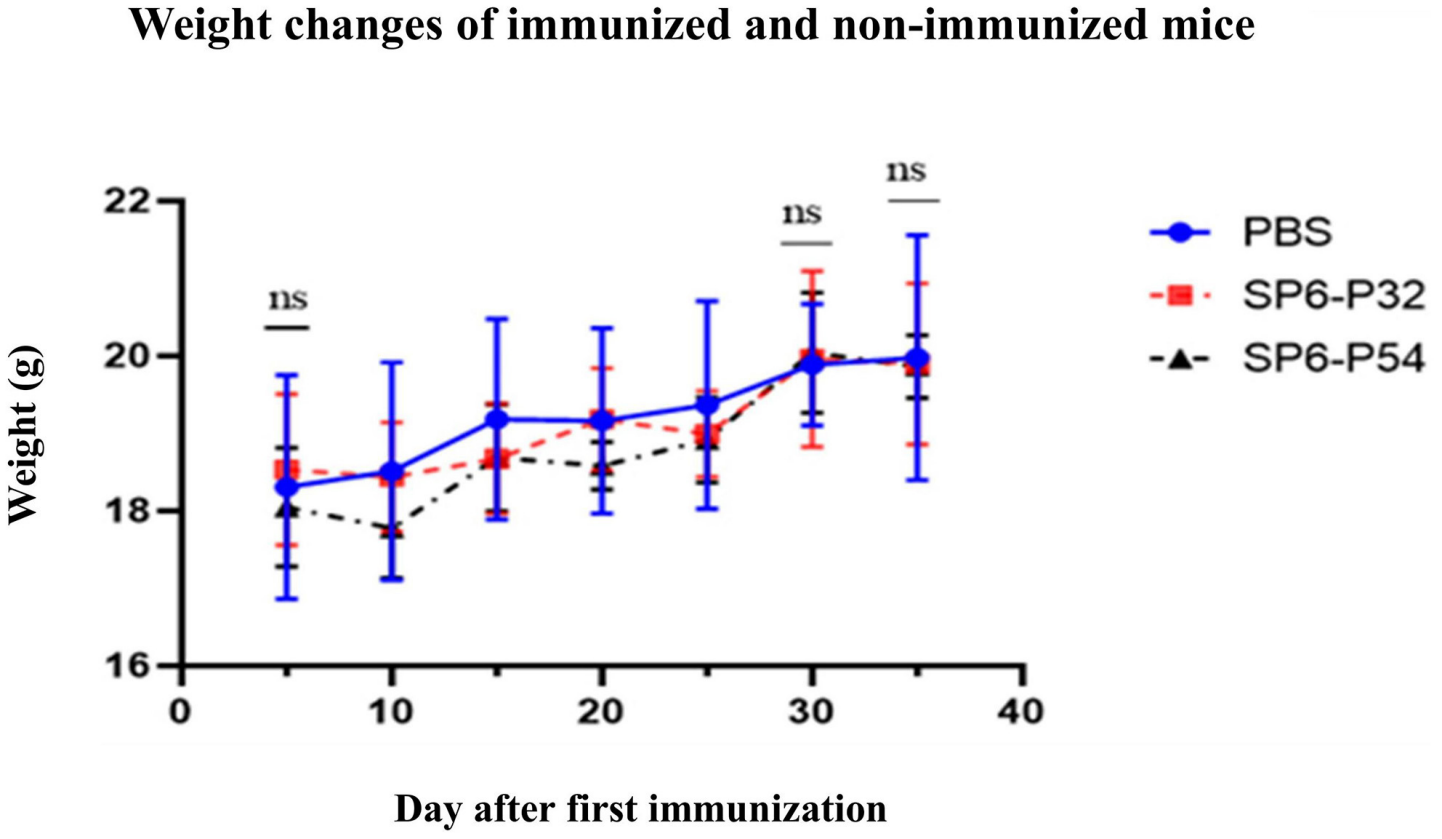
Comparative weight changes of immunized and non-immunized (PBS group) mice monitored for 35 days after the first immunization. ns, non-significant (*P* > 0.05).

As shown in Figure 6, the weight of the immunized group decreased slightly and began to increase gradually again 1 week later, while finally there was no difference in the weight and state of the mice compared to the PBS group after completing the third immunization. The result indicated there had no significant effect on the growth of mice after immunization with ASFV SFV-RPs.

### ELISA Results

**Figure 7:**
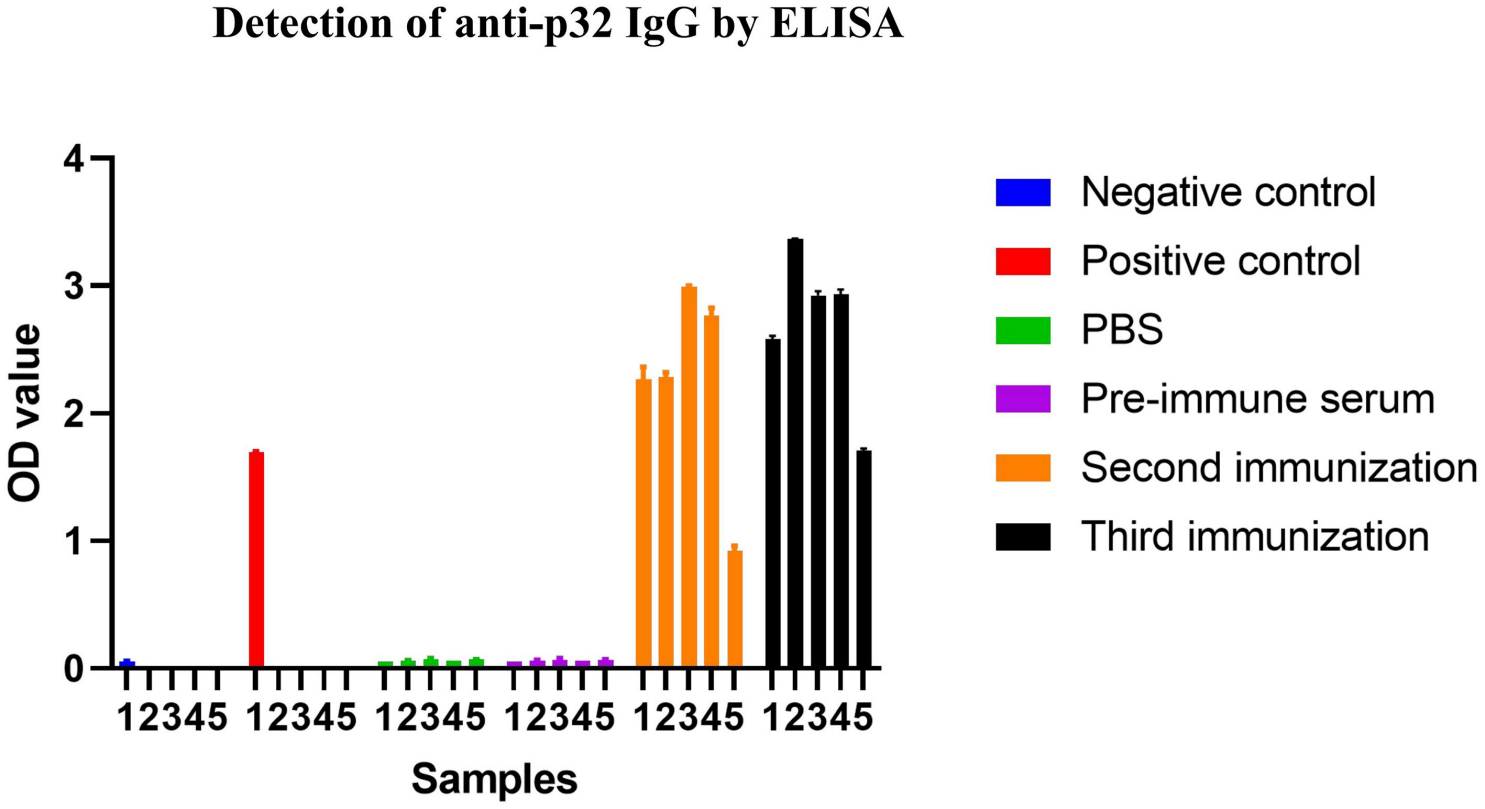
ELISA was used to detect anti-p32 IgG in each group. Cut off calculation: cut off = average value of negative control + 0.15. When the negative control value was ≤ 0.05, it was calculated as 0.05. Positive judgment: samples with OD value ≥ cut off were judged as positive. Negative determination: samples with OD value < cut off were judged as negative. SFV-p32 RP-immunized mice are numbered 1–5, which are consistent with the numbers assigned to mice detected by pre-immune serum, the second immunization and the third immunization in the FIGURE.

**Figure 8:**
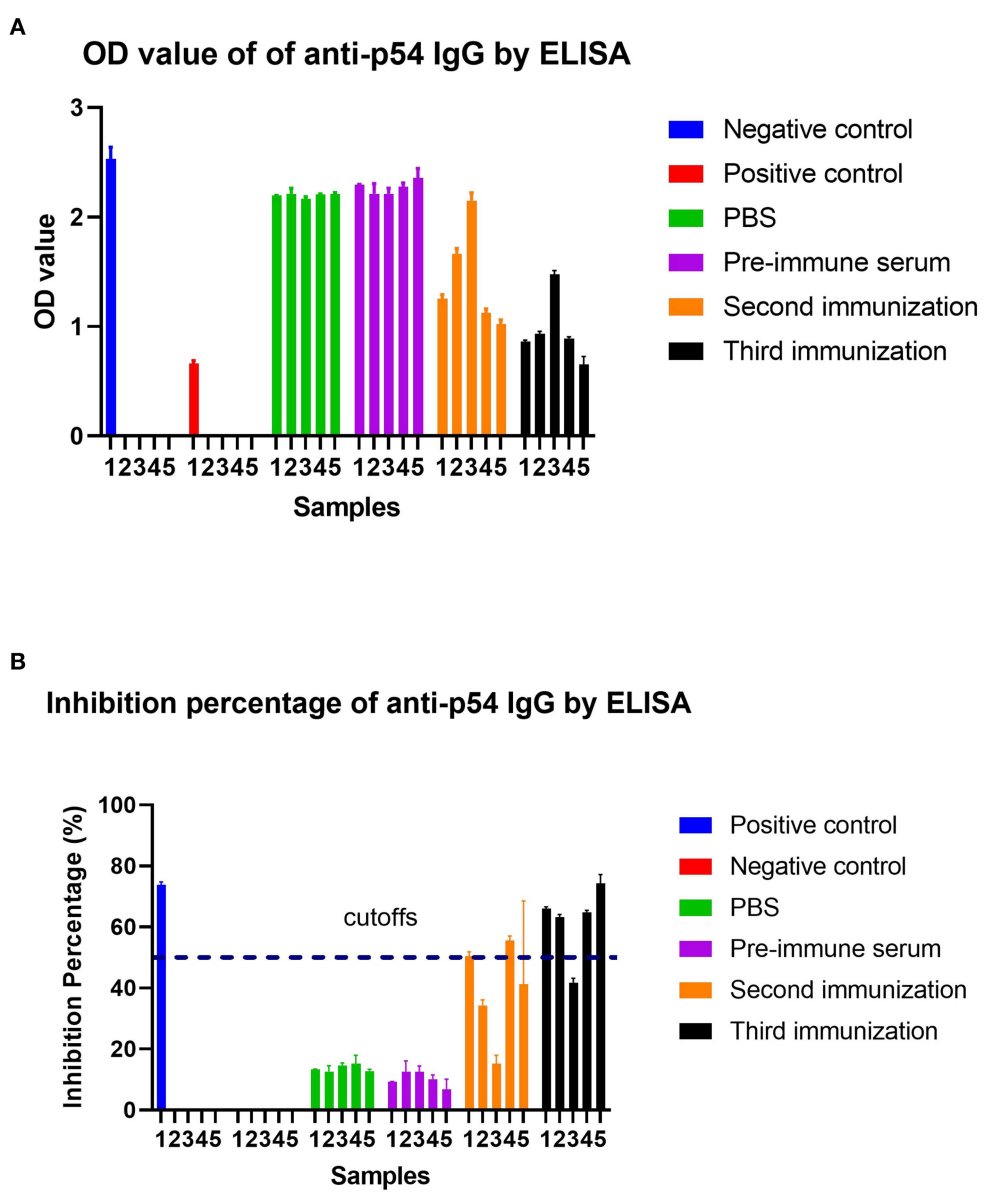
ELISA was used to detect anti-p54 IgG and calculate the inhibition percentage (IP) in each group. **(A)** ELISA results for each group. The mean OD of the negative control was ≥ 0.5. **(B)** Calculation of the IP of all samples in each group. IP of all samples was calculated as follows: IP (%) = (1–OD_450_ test sample/Mean OD_450_ negative control)*100. The IP (%) of the positive control was ≥55%. Serum/plasma samples were assessed as follows: IP ≤ 40% (negative), ASF antibodies were absent in the test samples; IP ≥ 50% ASF antibodies were present in the test samples; 50% > IP ≥ 40% doubtable, rechecked in 1–2 weeks. SFV-p54 RP-immunized mice are numbered 1–5, which are consistent with the numbers assigned to mice detected by pre-immune serum, the second immunization and the third immunization in the FIGURE.

Mice were immunized three times with SFV-p32 and SFV-p54 RPs. Serum collected 1 week after the second and third immunizations were tested using ELISA. As shown in Figure 7, SFV-p32 RP-infected mice had higher antibody levels after the third immunization compared to the second immunization. Similarly, SFV-p54 RP-infected mice produced IgG antibodies after the second and third immunizations (Figure 8). The results indicated that three immunizations induced robust antibody production.

### ELISPOT Results

**Figure 9:**
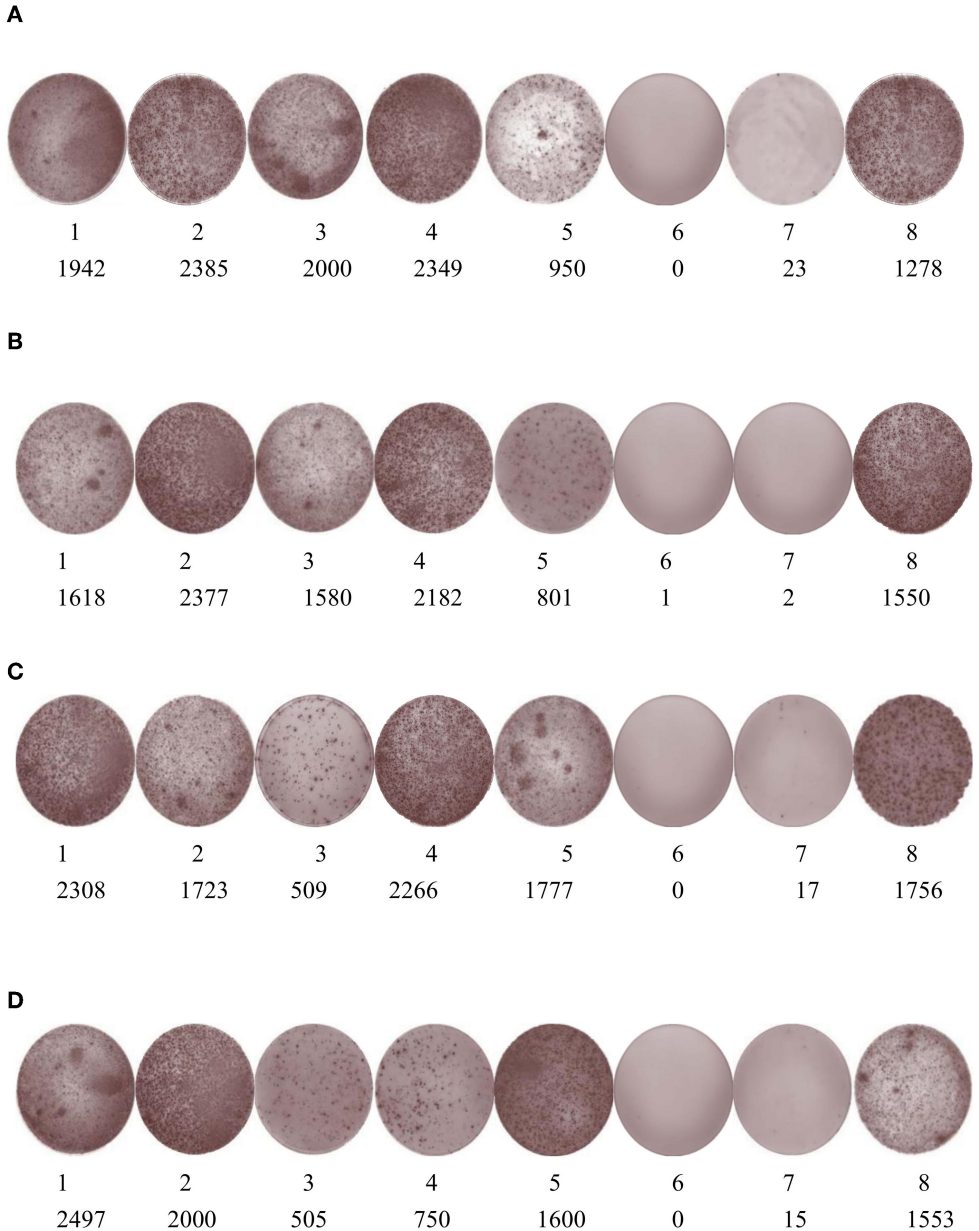
ELISPOT was used to detect IFN-γ and IL-4 expression in SFV-p32 and -p54 RP-immunized mice. **(A)** IFN-γ spots of SFV-p32 RP-immunized mice. **(B)** IL-4 spots of SFV-p32 RP-immunized mice. **(C)** IFN-γ spots of SFV-p54 RP-immunized mice. **(D)** IL-4 spots of SFV-p54 RP-immunized mice. For all four panels, 1–5 are the experimental groups and 6, 7, and 8 are the background control, negative control, and positive control, respectively. The numbers indicate the number of spots.

Two weeks after the third immunization, IFN-γ and IL-4 expression were detected in lymphocytes using ELISPOT (Figure 9). The results showed that SFV-p32 and -p54 RP-injected mice had higher expression of both cytokines compared to the negative and background controls.

## Discussion

Our results show that replication-defective viral particles can be produced by packaging SFV replicon RNA and first-generation helper RNA. The replication-defective viral particles were generated through amplification and recombination within ~24 h, with viral production peaking after 36 h. Due to the deletion of key sections of the SFV genome, the resulting replication-defective viral particles were only capable of performing one round of replication in infected cells (10). We initially produced SFVCs-EGFP RPs and found that over 50% of the cells expressed the EGFP.

Sjöberg et al. (14) indicated that the first 102 bases of the viral capsid gene play a role as a translational enhancer, and that SFV vectors that incorporate this RNA have improved heterologous protein synthesis. However, in the present study, the expression of the target protein was not reduced after the capsid-coding section was removed, which is different from previous results (14). We believe that the difference in results may be explained in several ways. Viral structural protein synthesis might be inhibited after SFV-infection so that the translation enhancer does not play a corresponding role. It was reported that the synthesis of viral structural proteins in extensive amounts upon Sindbis-infection engages <25% of the synthesizing capacity used by the cell for steady state protein production and host protein synthesis stops almost completely 5 h after infection (14, 15). Furthermore, it was reported that a mildly acidic pH may lead to the hydrolysis of the SFV capsid (16). In addition, it is unclear which C'- gene fragment enhances translation initiation and whether the effect results from an adaptation to the specific environment of SFV-infected cells (14). Further research is required to answer this question. In any case, to prevent the interference of the capsid protein on the specific expression of the target protein in subsequent animal experiments, the fused capsid protein should be removed. Therefore, we produced SFV-EGFP RPs. Finally, we produced ASFV SFV-RPs containing p32- and p54-encoding segments, which showed high levels of protein expression, implying that we were successful in establishing the SFV vector platform.

The serum contains numerous heterologous proteins, making it difficult to package RPs produced in cells for subsequent animal experiments. There is a need for safe and reproducible methods for vaccine development. In this study, we first used 1% FBS in the medium after co-electroporation. Once the majority of the BHK-21 had adhered, they were cultured in a serum-free medium (growth kinetic parameters including cell density, viability, maximum growth density, and viral titer are not shown in this paper). We found that this approach was helpful for the RP production, and even subsequent animal experiments. Other researchers have reported the merits of using serum-free media in the production of vaccines and other biological products (2, 17). In the future, we will optimize our culture media to support viral proliferation better.

In the present study, mice were immunized with SFV RPs containing ASFV antigens, which resulted in the robust induction of humoral and cell-mediated immune responses. Almost all mice inoculated with SFV-p32 and SFV-p54 (RPs) produced IgGs against the expressed antigen after the third immunization. Moreover, the levels of ASFV p32-specific IgG and ASFV p54-specific IgG remained high 5 months post immunization. The ELISPOT results showed that all immunized mice produced IFN-γ and IL-4, which indicates that the SFV-based vector vaccine induced a robust cell-mediated response. Although one mouse did not produce IgG antibodies after the third round of immunization with SFV-p54 RPs, it still exhibited a cellular immune response [Figure 9 (c-3, d-3)]. This might be because ASFV SFV-RPs are more effective at inducing cellular rather than humoral immune responses. ILs mediate T and B lymphocyte activation, proliferation, and differentiation during the activation and regulation of the cellular immune response. Our data demonstrate that SFV-p32 and SFV-p54 RPs also elicited high IL-4 expression in mice, an important mediator of the Th1-type response. There is evidence that cytotoxic CD8^+^ T lymphocytes play an important role in protection against ASFV, and cell-mediated response is critical for clearing the infection (5, 18).

The SFV-based vector vaccine is a defective single-cycle virus generated by substituting the structural protein-encoding genes from the SFV genome with the ASFV antigens and then electroporation this vector RNA into BHK-21 cells together with helper RNA. Numerous studies have documented the development of DNA and DNA replicon vaccines (19, 20). Although it was reported that DNA vaccines elicit humoral and cellular immune responses, they failed to protect animals from a virulent ASFV challenge (21). Moreover, RNA replicon vaccines based on the alphavirus replicon system have been evaluated in chickens, pigs, and humans, and provided complete protection against lethal challenges (4, 12). It was reported that young pigs exhibited a protective humoral immune response following influenza hemagglutinin (HA) mRNA RPs vaccination (4, 22). In addition, robust humoral and cellular responses were elicited in mice, rabbits, and rhesus macaques which were given HA and neuraminidase (NA) mRNA RPs vaccines (4, 23). Though Murgia et al. (5) already described ASFV RP-30 and RP-54 using VEEV, which were tested for expression levels in Vero cells and immunogenicity in swine, there is a lack of relevant research on the cellular immune response. The present paper is different from Murgia et al. (5) as it focused on the design and optimization of replicon particles and the study of cellular immune response, which we believe will be useful for further immunogenicity and protection experiments in pig models.

Recent strategies have involved the modification of the alphaviral vector 5′ terminal region to specifically identify cancer cells and induce their death (24). Although such research is still in its early stages, it holds promise for cancer treatment. Modified viruses can also be used in the research and development of cancer vaccines given the vast number of target proteins (25). Therapeutic alphaviral vector-derived cancer vaccines activate humoral and cellular immunity in the treatment of melanoma, as well as breast, mast cell, and prostate cancers (26). Despite the promise of viral vector vaccines, certain challenges remain (27). In this study, there was no recovery of infectious virus by blind passaging, IFA, or plaque assay; furthermore, no clinical symptoms of infection were observed in all inoculated animals, which indicate that use of this SFV vector system is valuable to express high levels of recombinant ASFV proteins and study the immunogenicity in pigs. It was reported that SFV-helper1 system is prone to generating the wild-type virus by recombination, however, using the pSFV-helper2 and the split helper can increase the safety of the system by decreasing recombination events (28–31). Therefore, in the next stage, we will use two other systems and compare them with the SFV-helper1 system to improve applications in clinical trials. In addition, there is still room for further alphaviral vector optimization, such as replacing the SP6 promoter with a cytomegalovirus (CMV) promoter, eliminating the *in vitro* transcription step, and minimizing RNA degradation, all of which could reduce costs and save effort.

## Conclusion

We established an SFV vector platform to produce replication-defective viral particles containing ASFV antigens, which induced effective antigen-specific humoral and cellular immune responses. The resulting RPs exhibited excellent biosafety and elicited a robust immune response due to the replication-defective nature of the viral particles and the use of a self-replicating RNA. However, in this study, we have not included any pig models. Nevertheless, we still believe that this paper will be useful for future immunogenicity research in pig models, providing a reference for the production of RNA replicon vaccines to elicit robust humoral and cellular immune responses.

## Data Availability Statement

The datasets presented in this study can be found in online repositories. The names of the repository/repositories and accession number(s) can be found in the article/supplementary material.

## Ethics Statement

The animal study was reviewed and approved by Animal Welfare and Ethical Censor Committee.

## Author Contributions

RC conceived the idea. NF did most of the experimental work and wrote the first draft of the manuscript. BY, HZ, and TX made the statistical analyses and did parts of the experimental work. YL assembled the data. HL and AZ advised in the process of manuscript writing. All authors contributed to the article and approved the submitted version.

## Funding

This study was supported by the Technology Planning Project of Guangdong Province (grant number 2019B020211004).

## Conflict of Interest

NF, BY, HZ, and RC were employed by Zhaoqing Institute of Biotechnology Co. Ltd. YL and RC were employed by Zhaoqing DaHuaNong Biology Medicine Co. Ltd. The remaining authors declare that the research was conducted in the absence of any commercial or financial relationships that could be construed as a potential conflict of interest.

## Publisher's Note

All claims expressed in this article are solely those of the authors and do not necessarily represent those of their affiliated organizations, or those of the publisher, the editors and the reviewers. Any product that may be evaluated in this article, or claim that may be made by its manufacturer, is not guaranteed or endorsed by the publisher.
